# Why do different people choose different university degrees? Motivation and the choice of degree

**DOI:** 10.3389/fpsyg.2014.01244

**Published:** 2014-11-13

**Authors:** Anya Skatova, Eamonn Ferguson

**Affiliations:** ^1^School of Psychology, University of NottinghamNottingham, UK; ^2^Horizon Digital Economy Research, University of NottinghamNottingham, UK

**Keywords:** motivation, choice of undergraduate degree, real life choices, undergraduate degree choice motivation, proximal motivation, prosocial motivation, intrinsic motivation

## Abstract

Different people choose undergraduate degrees to study at university for different reasons. To date, there have been limited attempts to identify individual differences in motivation that drive undergraduate degree choice. We identified that people choose university degrees for four reasons: career concerns (Career), intrinsic interest in the subject (Interest), an opportunity to help others (Helping) and because they are looking for an easy option to get into higher education (Loafing). We investigated whether these motivations apply to the choice of undergraduate degree in two samples: (1) undergraduate (*N* = 989) and (2) prospective (*N* = 896) students. We developed the Motivations Influencing Course Choice (MICC) questionnaire to measure these motivations. Scales of Helping, Career, Loafing, and Interest showed good psychometric properties, showed validity with respect to general life goals and personality traits, and predicted actual and prospective degree choices. We demonstrated that medical degrees were chosen due to a mixture of Helping and Career, while engineering degrees were associated with Career and low Interest in the degree. The choice of arts and humanities degrees was driven by Interest and low concern about future career, accompanied with high Loafing. We also demonstrated gender differences: females were high in Helping (both samples) and Interest (only in the undergraduate sample) motivation, while males scored higher in Career (only in the undergraduate sample) and Loafing (both samples). The findings can feed into both theoretical accounts of proximal motivation as well as provide help to improve degree programmes at universities and support better career advice.

## Introduction

Motivations have been shown to affect real life outcomes: pro-social motivation predicts volunteering, while motivation of achievement predicts career success (Judge et al., 1995; Carlo et al., 2005). Each motivation corresponds to the subjective reward one expects to get from the chosen activity (e.g., pro-social motivation is associated with rewards from helping others), and those expected rewards guide people's choices. However, most real life choices bring multiple rewards (e.g., volunteering can be chosen to help others, because it is intrinsically rewarding as well as because it might help in certain career paths). Therefore, to predict choices we need to account for multiple motivations. Here we use the term motivation as broadly associated with understanding of the “why” of human behavior (Deci and Ryan, 2000). This paper focuses on individual differences in motivation leading to the choice of undergraduate degrees.

The choice of undergraduate degree is likely to be one of the first major life decisions young people take. Understanding factors that affect this decision is important in helping to provide more efficient career advice and help to tailor educational programs to students' expectations. For example, if career services identify that a student is looking for a degree that is intrinsically rewarding, they can recommend degree types that may satisfy that need. This might secure a better match between degree types and students and reduce drop-out rates.

Along with external pressure from parents and schools, the choice of undergraduate degree is affected by multiple psychological factors, including person's own motivations for further education as well as what they expect to get out of their degree. Disciplines offer different opportunities to fulfill these motivations and that, in part, defines the choices people make. For example, those who are career minded are potentially more likely to choose a degree with clear career prospects, while others might be motivated purely by intrinsic motivation for their subject, whether or not it has clear career prospects or earning potential. Previous research on motivation related to career choice has mostly focused on variables like indecisiveness (Guay et al., 2006) and achievement motivation (Collins et al., 2004; Watt and Richardson, 2007). These variables were used to predict how successful a student is in making their choice or how successful they are in performing in their degree, respectively. Whilst it is possible that students who have chosen different degrees could differ in the levels of indecisiveness or achievement motivation, by their own definition these constructs do not aim to predict whether a student chooses, for example, arts or sciences. Therefore, establishing motivational differences that predict the choice of undergraduate degree will contribute to understanding why different people make different decisions in this domain.

There is a rich research tradition examining psychological variables that are relevant to understanding of undergraduate degree choice motivations. The most influential theory in vocational research (Holland, 1985) equates chosen career path to the reasoning behind the choice. For example, it has been demonstrated that social vocational interests are associated with the choice of medicine (Holland, 1985). However, those choosing medicine might not only strive to help others but it is possible that they make the choice because of career opportunities, too. Therefore, suggesting that there is a dominant motivation for each career path overlooks the complexity of motivations that are involved in undergraduate degree choice decision.

The role of individual differences in motivation is important in predicting choices. The principles of multidetermination proposed by Pervin (2001) suggest that any choice is a result of a combination of motivations. This highlights the importance of both differences in motivation and what kind of outcomes those differences predict. Surprisingly little work has been done to study individual differences in motivations for university degree choice, with some investigations focusing on specific degrees (e.g., sciences or engineering degrees, Deemer et al., 2010; Savage et al., 2012), but not relating generic, non-specific degree reasons to motivational dimensions such as intrinsic motivation.

Previous research demonstrated generic differences in motivation such as pro-social, achievement or intrinsic motivation (Heckhausen, 1977; van Lange et al., 1997; Deci and Ryan, 2002). An important feature of these motivational constructs is that they claim, according to respective theoretical accounts, to predict real life outcomes in a variety of domains. For instance, intrinsic motivation has been shown to predict performance in sport, health, education, work, and other domains (Kasser and Ryan, 1993; Conroy and Elliot, 2004; Walker et al., 2006; Conroy et al., 2009; Hagger and Chatzisarantis, 2009). It is not clear whether these generic motivational differences are applicable to explain individual differences in undergraduate degree choice reasons.

This study, therefore, employed motivation constructs to understand undergraduate degree choice motivation. The research presented here builds on previous literature by examining whether general motivational dimensions, such as pro-social and achievement motivation, extend to predict the choice of undergraduate degree. In order to develop a model of individual differences in motivation for undergraduate degree choice, four generic motivations were identified from the literature that are seen as relevant to the context of choice of undergraduate degree (see Skatova, 2011 for more details).

### Helping motivation

First, we examined a *helping* or pro-social motivation: a preference to work with people and engage in activities that benefit society. Helping motivation drives behaviors which benefit the community, society overall and/or other individuals. Pro-social motivation predicts more active engagement in volunteering activities (e.g., Cameron et al., 1998; Carlo et al., 2005) and monetary donations to charities (e.g., Van Lange et al., 2007). Pro-social motivation is described in the vocational domain as social vocation interest (Tracey and Rounds, 1996) or within the general life aspirations as community life goal (Kasser and Ryan, 1996).

### Career motivation

Second, we examined a *career* motivation or striving for external success, which combines properties of both extrinsic and achievement motivation. Extrinsic motivation is defined as being driven by external rewards (Deci and Ryan, 2002), while achievement motivation implies striving to excel compared to a reference group. Achievement motivation has been shown to predict real life outcomes in education, sport, work, and other domains (Conroy and Elliot, 2004; Porath and Bateman, 2006; Vansteenkiste et al., 2007; Richardson and Abraham, 2009). Career motivation was previously related to vocational choices as a dimension of prestige (Tracey and Rounds, 1996).

### Interest or intrinsic motivation

Third, we studied intrinsic motivation which reflects inherent interest in the activity and is defined by the expectation of enjoyment from the activity (Deci and Ryan, 2002). An activity is intrinsically motivated when it is performed for the sake of the pure enjoyment of the process (Deci, 1971). Intrinsic motivation is known to guide choices when goals have a personal internalized significance for an individual. Self-Determination Theory (SDT, Deci, 1971) suggests that a propensity to be involved in more or less intrinsically motivated activity is a disposition, which can explain individual variation in terms of choice outcomes in different domains (Neighbors and Larimer, 2004; Gagné and Deci, 2005; Lam and Gurland, 2008; Komarraju et al., 2009). Studies provide evidence that intrinsic, as compared to extrinsic motivation, predicts higher life satisfaction, self-esteem, and self-actualization; attenuates depression and anxiety, and also provides an increase in cooperative behavior (Kasser and Ryan, 1993, 1996; McHoskey, 1999; Sheldon and McGregor, 2000; Sheldon et al., 2000; Vansteenkiste et al., 2007). Walker et al. (2006) showed that in educational settings intrinsic motivation predicts a choice to seek for a meaningful cognitive engagement (a productive way of learning, when one tries to understand concepts rather than memorize information).

### Loafing motivation

Finally, loafing represents a strategy to engage in activities to provide the easiest route to one's goals, which could be also related to degree choice. In behavioral economics literature similar behavior is referred to as free-riding or self-regarding behavior that results in exploiting communal resources without equivalent compensation (Carpenter, 2007). Free-riding is linked to a broad cluster of social phenomena, including social loafing (Arnscheid et al., 1997) and anti-social behaviors (Nikiforakis, 2008). It is employed to explain tax avoidance, causes of financial crises and the development of society in general (Boadway et al., 2007; Dabrowski, 2010). Research in the vocational domain highlights the role of this “dark-side” of human motivation in respect to career success (Kasser and Ryan, 1993; Furnham et al., 2013). Social loafing (i.e., exploiting communal benefits without proportional contribution into communal good, Karau and Williams, 1993) could be potentially relevant to the choice of a university degree. Free-riding as an individual strategy have been primarily studied within behavioral economics, and it is still unclear if it generalizes to other behavioral domains, such as career or degree choice.

### Development of undergraduate degree choice motivation measure

To investigate the motivations for degree choice and their relationships to actual choices, we need to be able to measure differences in motivation. Of the existing psychometric tools that could be used to assess degree choice motivation [e.g., Aspiration Index (Kasser and Ryan, 1996), Vocational choice: Self-Directed Choice Inventory (Holland, 1985), Research Motivation Scale (Deemer et al., 2010), or Personal Globe Inventory (Tracey, 2002b)], none measure university degree choice motivations directly. Further, loafing is missing from all existing measures. Therefore, the aim of this paper was to develop and validate a new tool to assess helping, career, loafing and interest motivations for university degree choice: Motivations Influencing Course Choice (MICC) questionnaire.

### Validation of degree choice motivation dimensions

To demonstrate validity of the new measure we used the Aspirations Index (Kasser and Ryan, 1996). The Aspiration Index provides a measure of general life goals (both intrinsic and extrinsic), which is a proxy for motivation, and therefore Aspiration Index scales (intrinsic: Relationships, Community, Personal Growth; extrinsic: Wealth, Fame, Image) can be used to test validity of the MICC questionnaire. Further, as multiple links between motivation and personality have been previously reported in the literature, the validity of the proposed model was assessed in relation to the Five Factor Model (Goldberg et al., 2006).

#### Helping

Altruism, which implies an aspiration to help others, is a facet of agreeableness, thus helping motivation should be associated positively with agreeableness (Carlo et al., 2005; Penner et al., 2005). Further, helping encompasses concern for others, therefore, it is conceptually close to life goal Community and should be positively associated with helping.

#### Loafing

The loafing orientation implies a tendency to reach one's goals via the easiest route, even if that involves exploiting others. In part, extraversion is an orientation toward others (Ashton et al., 2002). Therefore, low extraversion implies a reduced of consideration of others' welfare and should be related to loafing. Conscientiousness implies a thorough attitude toward one's work and should be negatively associated with loafing (Mohammed and Angell, 2003; Hoon and Tan, 2008). Further, loafing, as it implies concern about oneself, should be related to self-oriented extrinsic life goals from Aspiration Index: Wealth, Fame and Image.

#### Career

It has been shown that both extraversion and conscientiousness are associated with career motivation and career success (Judge et al., 1999; Seibert and Kraimer, 2001; Komarraju et al., 2009). One of the major indices of career achievements is financial success. Therefore, career motivation should be positively linked to conscientiousness, extraversion and Wealth.

#### Interest

Intrinsic motivation in job settings was previously shown to be positively related to conscientiousness and openness (Furnham et al., 2002). Thus, the interest motivation should be positively associated with conscientiousness and openness from the Big Five, and Personal Growth, central facet reflecting intrinsic motivation in the Aspiration Index scale.

#### Construct validity

To investigate the construct validity of the undergraduate degree choice motivations their associations with actual degree choices were tested. Below we discuss predictions with regards to the undergraduate degree choices and motivation types.

Both medical and social sciences are conceptualized as being a social type vocations (Holland, 1985) with those who pursue them reporting higher social vocational interests (de Fruyt and Mervielde, 1996; Crossley and Mubarik, 2002). Helping implies being oriented toward others and preference for working with people. Therefore, we expected that choices of both medical and social sciences degrees to be positively predicted by helping. Science and engineering degree students, contrary to that, report lower social vocational interests (de Fruyt and Mervielde, 1996). Therefore, we expected that the choice of both science and engineering degrees was negatively predicted by orientation toward helping.

Loafing orientation implies taking an easy option. In the degree choice domain, loafing implies pursuing an easy degree that does not require much effort. Science and engineering degrees are often seen as relatively labor-intense options that might require a lot of thorough effort. Therefore, we expected the choice of science and engineering degrees to be negatively predicted by loafing.

It has been shown that arts and humanities students report artistic vocational interests (which is conceptually similar to intrinsic motivation, de Fruyt and Mervielde, 1996). In addition, science students report that intrinsic rewards are important in their choice of degree (Deemer et al., 2010), while engineering students report low artistic vocational interests (de Fruyt and Mervielde, 1996). We, therefore, expected interest to positively predict arts and humanities and sciences and negatively predict engineering.

Finally, an often reported reason to choose medical or engineering degree is to be successful in one's career (Inkson, 1971; Tracey, 2002a). Therefore, we expected career orientation to positively predict the choice of medical and engineering degree.

#### Incremental validity

To demonstrate incremental validity of the new scales, we tested if they predicted actual undergraduate degree choices over general life goals, measured by Aspiration Index. The Aspiration Index reflects general life goals but does not measure specific reasons behind specific choices, such as undergraduate degree choice. Thus, we expected that degree choice motivation scales should generally correlate with respective Aspiration Index scales, and interest, career, helping, and loafing measure of undergraduate degree choice motivation to be a stronger predictor of the actual choices of specific degrees. It is plausible that a person is generally community oriented and wishes to help others, but they choose their undergraduate degree for good career opportunities. Further, it has also been shown that life goals are highly correlated with each other (Kasser and Ryan, 1996), suggesting that there may be fewer underlying dimensions of general life goals than proposed previously. If a more parsimonious model with four factors could predict better real life choices, it would be both theoretically and empirically beneficial. We tested incremental validity of the model by dividing undergraduate degree types into arts and humanities, engineering, social science, medical sciences, and sciences. This division represent a common degree division in UK universities.

#### Gender

In addition we also investigated gender differences in helping, career, interest, and loafing. Previous studies report greater intrinsic motivation in women, greater extrinsic motivation in men, as well as greater commitment to social values in women (Ferssizidis et al., 2010). We, therefore, predicted that men endorse career goals more than women, and women express higher interest and helping motivation.

## Materials and methods

### Participants and procedures

We developed a measure of university degree choice motivation on a sample of university undergraduates (Sample 1) and replicated the measure in a sample of prospective university students who attended an Open Day (Sample 2). We used both samples to test the validity of the new developed scales. The study was approved by Ethics Committee of the School of Psychology, University of Nottingham.

Sample 1 data was collected through an online and pen/paper form of the survey. An opportunity sample of 989 participants from two large UK universities was recruited (mean age 20.48, range 17–49, 42.5% male, 46.5 % of the sample were in their first year of university, 23.8%, second, 29.6%, third or higher), incentivised by a prize draw of £75. The overall sample included three opportunity subsamples: an online from a large city-based British university (“Online 1,” *N* = 397), an online from a large campus-based British university (“Online 2,” *N* = 272) and a pen/paper from the same university as a second sample (“P&P,” *N* = 271). Subsamples differed significantly in age and gender, with participants from the subsample P&P being younger than both Online 1 and Online 2 [*F*_(2, 937)_ = 22.87, *p* < 0.001]. Subsample P&P had equal gender distribution (46.9% female); proportion of females was significantly higher in Online 1 and Online 2 [59.9 and 64.7% female, respectively, χ^2^(2) = 19.32, *p* < 0.001]. The overall sample was not different in gender (57.5% female) and age (*M* = 20.48, *SD* = 2.8) from a typical undergraduate sample. As subsamples differed in age and gender, we controlled for these variables in all subsequent analyses. Some participants did not fill in all of the questionnaires (they were free to withdraw at any point). Participants who did not finish all questionnaires were included in all relevant analyses (where it was possible) as they were not different in characteristics from the rest of participants. 3.3% of the overall sample reported studying medical sciences, 29.1% social sciences, 44.7% sciences, 11.1% arts and humanities and 11.8% engineering.

Sample 2 data was collected online by distributing the study link to a mailing list of university open day attendees. An opportunity sample of 896 participants responded to the survey (mean age 17.36 years, range 16–47, 28.1% male), with an incentive of a prize draw of £75 in Amazon vouchers. 16.2% of the overall sample reported wishing to study medical sciences, 25.8% social sciences, 24.9% sciences, 21.9% arts and humanities and 3.1% engineering.

### Motivations influencing course choice: item generation

MICC was developed to measure different undergraduate degree choice motivations. Items were generated corresponding to four themes described in the introduction: helping, career, interest, and loafing. All items were answered on 6-point Likert-type scales with participants judging how relevant each specific reason was for their choice of degree, ranging from *not at all* to *very much so*. Two pilot studies were conducted with opportunity samples of undergraduate students (*Ns* = 115 and 187), which aimed to ensure that students could relate to the content of the items. Out of initial pool of fifty-five, six items were removed due to a high skew. The full initial list of items is reported in Supplementary Materials. In the undergraduate sample we asked participants about their reasons for undergraduate degree choice using stem “I have chosen this degree because…,” followed by respective reason, e.g., “I was interested in the subject,” while for the open days sample the stem was changed to “I am choosing this degree because…”

### Factor analytic procedures

Sample 1 was randomly split in half, with one half used for Exploratory Factor Analysis (EFA, *N* = 494) and the second for Confirmatory Factor Analysis (CFA, *N* = 495). Comprehensive Exploratory Factor Analysis (CEFA) (Browne et al., 2008) was used to estimate the EFA. CFA models were estimated with Sattora-Bentler correction in LISREL 8.7 and ran from polychoric correlations estimated from the asymptotic covariance matrix (Du Toit et al., 1999). Model fit was assessed using the χ^2^-value, the root mean square error of approximation (RMSEA), the comparative fit index (CFI) and the incremental fit index (IFI). A model with a RMSEA below 0.08 and CFI and IFI greater 0.95 indicates a good fit of the data (Hu and Bentler, 1999). We ran only CFAs on Sample 2.

### Validity and reliability

Reliability of the scales was assessed using Cronbach alphas and mean inter-item correlations. Validity of the MICC scales were assessed through associations with the Aspirations Index (Kasser and Ryan, 1996) and the Big Five. The stem for each item in the Aspiration Index was “How important is this to you?” and response options range from not at all (1) to very (7). It included six scales (Relationships, Community, Personal Growth, Wealth, Fame, Image) with five items in each scale. Goldberg's 35 bi-polar markers (Goldberg et al., 2006) were used to measure the Big Five, including Extraversion, Agreeableness, Conscientiousness, Emotional Stability, and Openness scales. Respondents rated adjectives on a 9-point Likert-type scale from *very inaccurate* (1) to *very accurate* (9).

## Results

### CEFA

Prior to the analyses, one item was removed due to a high skew. Parallel analysis on the remaining forty-eight items confirmed a four factor structure (Turner, 1998). After several iterations of CEFA, items were removed as follows: six items did not load significantly on any of four factors, sixteen items loaded significantly on more than one factor; eight items had very similar content to the items that were kept in the questionnaire. Parallel analysis on the eighteen remaining items confirmed four factors which corresponded to the predicted theoretical model, and explained 60% of variance with good fit statistics (based on CEFA): χ^2^ = 403.54, *df* = 87, *RMSEA* (95% *CI*) = 0.086 (0.078; 0.094). The remaining four factors all had at least three items with loadings >0.57. These four factors were identified as follows: Helping, Loafing, Interest, and Career (see Table 1 for items, their means, standard deviations, as well as factor loadings).

**Table 1:** **Descriptive statistics for the final version of the MICC scales (Sample 1, undergraduate students)**.

	**Helping**	**Loafing**	**Interest**	**Career**	**Mean**	***SD***
02. I want to help other people.	**0.79**	−0.03	0.11	0.07	3.79	1.52
09. I want to serve society.	**0.73**	−0.03	0.05	0.09	3.51	1.43
12. I am interested in people.	**0.67**	0.07	0.18	0.01	3.83	1.55
18. I want to make the world a better place.	**0.61**	−0.05	0.14	−0.01	3.64	1.59
17. I am interested in understanding other people's perspectives.	**0.54**	0.13	0.20	−0.08	3.44	1.61
16. The degree seemed to be easy to pass.	0.04	**0.84**	−0.12	−0.07	1.81	1.13
14. I knew that I'd manage to pass the degree without doing too much work.	0.00	**0.79**	−0.12	0.04	1.90	1.20
06. It was the easiest option for me.	−0.06	**0.57**	−0.13	0.02	2.22	1.34
03. I'm not particularly concerned about other people.	−0.26	**0.34**	−0.06	−0.01	1.76	1.15
05. My individual goals are more important than the prosperity of society.	−0.09	**0.31**	0.03	0.19	2.51	1.37
13. It is a fascinating subject to study.	0.13	−0.14	**0.83**	0.03	4.74	1.24
11. For me it is very important to study a degree that I enjoy.	0.17	−0.02	**0.77**	−0.02	4.87	1.24
04. I wanted to know more about this subject.	0.10	−0.19	**0.73**	−0.01	4.91	1.16
01. I was always interested in this subject.	−0.02	−0.01	**0.60**	0.02	4.73	1.24
15. It provides me with secure career options.	0.00	−0.02	−0.03	**0.85**	4.07	1.47
10. It provides good career options.	0.07	−0.08	0.09	**0.81**	4.52	1.31
07. I want to get a well-paid job in the future.	0.02	0.06	−0.09	**0.70**	4.29	1.47
08. It is very competitive and I am an achiever.	0.12	0.19	0.09	**0.50**	3.40	1.54

All items in bold load significantly on the corresponding factors (p < 0.01).

### CFA

**Figure 1:**
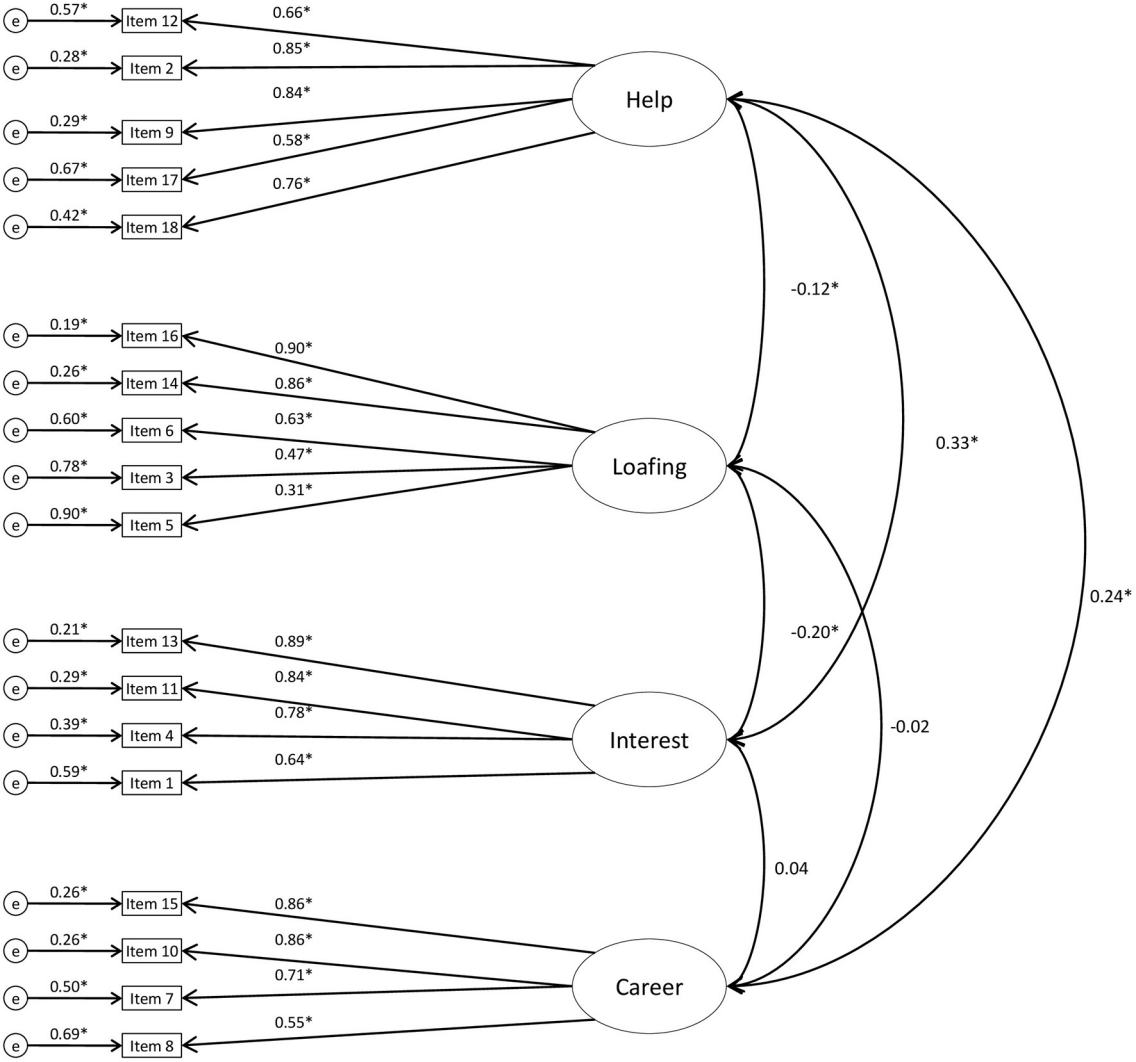
**CFA model with standardized estimates: MICC, Sample 1 (*N* = 495)**.

**Figure 2:**
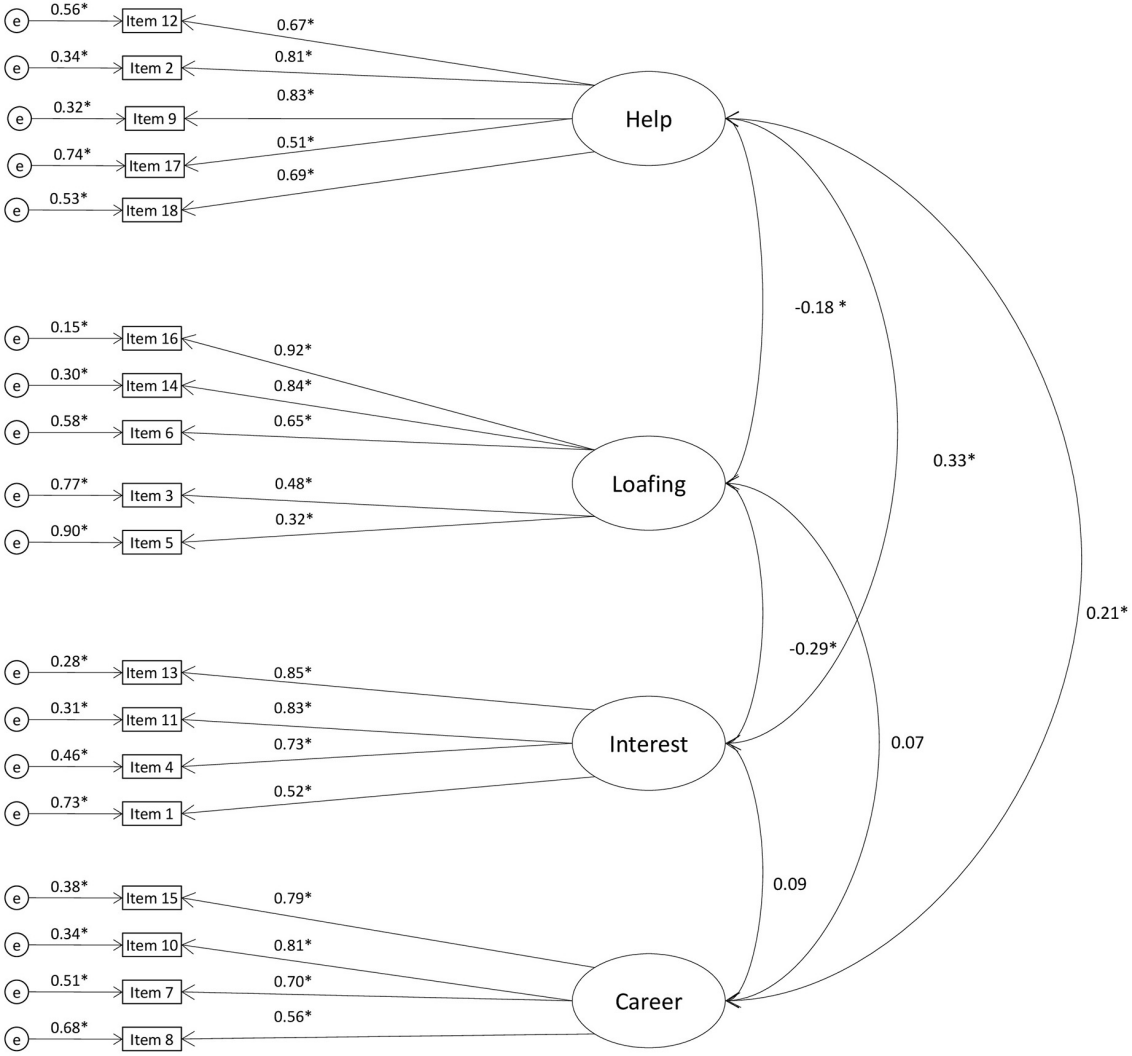
**CFA model with standardized estimates: MICC, Sample 2 (*N* = 896)**.

The four-factor oblique model, based on the CEFA, demonstrated good fit to Sample 1 (see Figure 1) data [χ^2^ = 434.94, *df* = 129, *p* < 0.001, *RMSEA* (95% *CI*) = 0.069 (0.062; 0.077), *CFI* = 0.95, *IFI* = 0.95, *N* = 495] and to Sample 2 (see Figure 2) data [χ^2^ = 759.71, *df* = 129, *RMSEA* (95% *CI*) = 0.074 (0.069; 0.079), *CFI* = 0.94, *IFI* = 0.94, *N* = 896].

### Reliability and descriptives

Correlations between scales, Cronbach alphas, and mean inter-item correlations (MICs) are shown in Table 2. Cronbach alphas for all subscales were good and demonstrated that the scales were internally consistent: mean Cronbach alpha was 0.80 for Sample 1 (*SD* = 0.05; range = 0.72–0.84), and 0.80 for Sample 2 (*SD* = 0.05; range = 0.72–0.84). With regard to scale homogeneity, the MICs were acceptable (mean = 0.48, *SD* = 0.07; range = 0.34–0.57 for Sample 1, and mean = 0.43, *SD* = 0.06; range = 0.33–0.50 for Sample 2) for subscales to measure relatively narrow constructs (Clark and Watson, 1995).

**Table 2:** **Correlations, descriptives (mean and *SD*), Cronbach alphas and mean inter-item correlations (MICs) for Sample 1 (S1, *N* = 989) and Sample 2 (S2, *N* = 896)**.

		**1**	**2**	**3**	**4**	**Mean (*SD*) of each scale**	**MIC**	**Cronbach alphas**
Helping (1)	S1	1	–	–	–	3.63 (1.19)	0.49	0.83
	S2	1	–	–	–	3.79 (1.15)	0.50	0.80
Loafing (2)	S1	−0.07^*^	1	–	–	2.08 (0.85)	0.34	0.72
	S2	−0.16^**^	1	–	–	1.80 (0.68)	0.33	0.71
Interest (3)	S1	0.26^**^	−0.13^**^	1	–	4.48 (0.99)	0.57	0.84
	S2	0.26^**^	−0.12^**^	1	–	5.46 (0.65)	0.43	0.75
Career (4)	S1	0.12^**^	0.11^**^	0.05	1	4.03 (1.14)	0.53	0.82
	S2	0.13^**^	0.17^**^	0.06	1	4.22 (1.05)	0.47	0.78

Significant simple zero-order correlations shown in bold:

** p < 0.01,

* p < 0.01.

**Figure 3:**
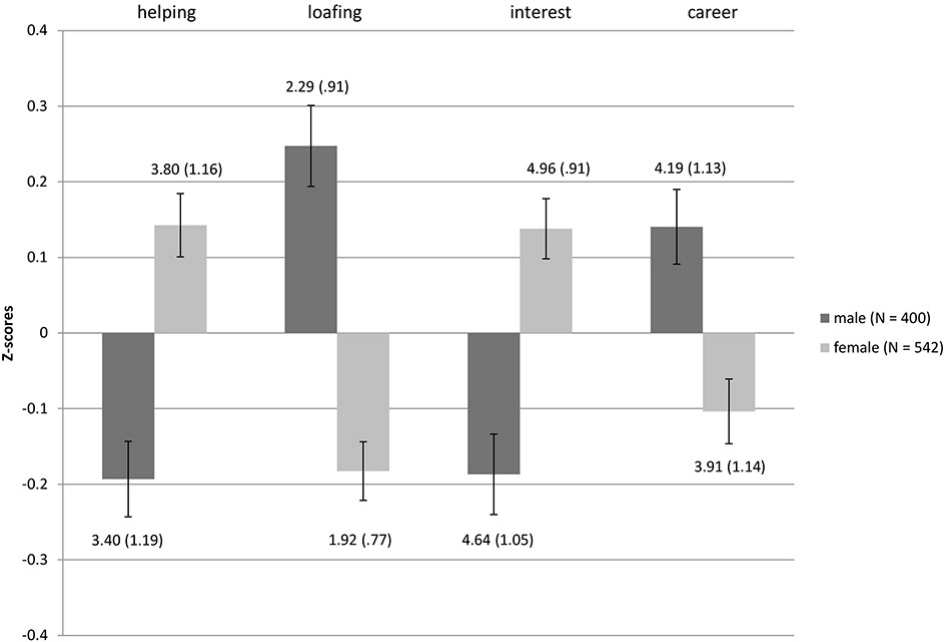
**Differences in MICC motivations between genders for Sample 1**. Bars represent the mean standardized scores and error bars represent the standard error of the mean. Values represent the means with standard deviations in parentheses.

**Figure 4:**
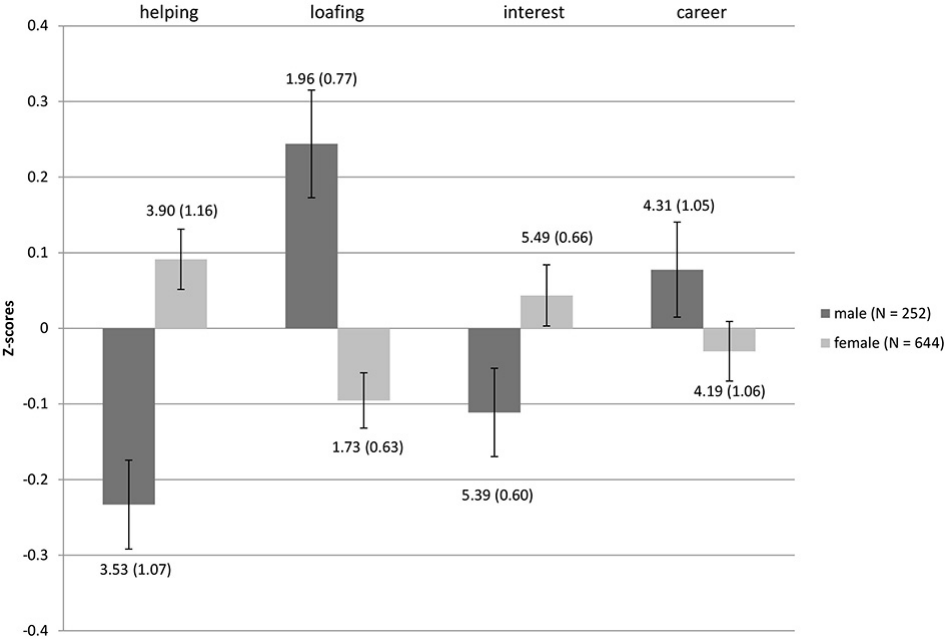
**Differences in MICC motivations between genders for Sample 2**. Bars represent the mean standardized scores and error bars represent the standard error of the mean. Values represent the means with standard deviations in parentheses.

Gender differences in motivations to choose undergraduate degrees were consistent with those reported previously in the literature in both samples (see Figures 3, 4 for mean standardized scores, as well as mean raw scores and standard deviations): females were higher in Helping motivation [*t*_(940)_ = −5.16, *p* < 0.0001 Sample 1, *d* (95% *CI*) = 0.34 (0.21; 0.47); *t*_(894)_ = −4.410, *p* < 0.0001, *d* = 0.33 (0.18; 0.47) for Sample 2], while males were higher in Career [*t*_(940)_ = 3.73, *p* < 0.0001, *d* = 0.25 (0.12; 0.38) only Sample 1]. Females in both samples assigned more importance to Interest in their degree concerns [*t*_(940)_ = −4.99, *p* < 0.0001, *d* = 0.33 (0.20; 0.46) Sample 1, *t*_(894)_ = −2.09, *p* < 0.05, *d* = 0.16 (0.01; 0.30) Sample 2], while choosing what to study compared to male participants. In addition, in both samples males were higher in Loafing [*t*_(940)_ = 6.67, *p* < 0.0001, *d* = 0.45 (0.32; 0.58) for Sample 1, *t*_(894)_ = 4.62, *p* < 0.0001, *d* = 0.34 (0.20; 0.49) for Sample 2]^1^.

### Convergent validity of the MICC scales

Zero-order correlations in both samples between the MICC motivations, Aspiration Index scales and the Big Five traits are presented in Table 3.

**Table 3:** **Cronbach alphas, means, standard deviations for personality traits and life goals, and their zero-order correlations with the MICC scales in Sample 1 (S1, *N* = 989) and Sample 2 (S2, *N* = 896)**.

				**MICC subscales**			
		**Mean (*SD*)**	**Alpha**	**Helping**	**Loafing**	**Interest**	**Career**
**BIG FIVE**							
Extraversion	S1	6.12 (1.27)	0.82	**0.10**	**−0.10**	0.06	0.08
	S2	6.20 (1.33)	0.83	**0.16**	**−0.14**	**0.12**	**0.10**
Agreeableness	S1	6.86 (1.09)	0.78	**0.19**	**−0.22**	**0.10**	**0.13**
	S2	6.98 (1.05)	0.77	**0.27**	**−0.30**	**0.21**	0.04
Conscientiousness	S1	6.44 (1.26)	0.81	**0.18**	**−0.21**	**0.21**	**0.16**
	S2	6.71 (1.14)	0.78	**0.13**	**−0.21**	**0.22**	**0.10**
Emotional stability	S1	5.62 (1.31)	0.80	0.04	0.02	0.03	**0.11**
	S2	5.67 (1.29)	0.80	0.06	**−0.12**	**0.11**	**0.09**
Openness	S1	6.94 (0.99)	0.74	**0.15**	−0.05	**0.20**	**0.12**
	S2	7.05 (0.94)	0.72	**0.18**	−0.09	**0.23**	**0.10**
**LIFE GOALS**							
Wealth	S1	4.39 (1.39)	0.90	−0.09	**0.20**	**−0.11**	**0.48**
	S2	4.21 (1.42)	0.91	**−0.22**	**0.29**	**−0.11**	**0.47**
Fame	S1	3.42 (1.29)	0.85	**0.14**	**0.29**	−0.02	**0.19**
	S2	3.19 (1.29)	0.86	0.03	**0.21**	−0.06	**0.21**
Image	S1	3.49 (1.38)	0.86	0.09	**0.22**	**−0.09**	**0.24**
	S2	3.40 (1.37)	0.88	0.03	**0.18**	−0.05	**0.26**
Personal growth	S1	5.85 (0.85)	0.74	**0.30**	**−0.21**	**0.32**	**0.15**
	S2	5.98 (0.79)	0.72	**0.25**	**−0.14**	**0.33**	**0.15**
Community	S1	5.12 (1.23)	0.90	**0.63**	**−0.24**	**0.22**	−0.01
	S2	5.21 (1.19)	0.87	**0.59**	**−0.29**	**0.23**	0.003
Relationships	S1	6.23 (0.98)	0.81	**0.14**	**−0.26**	**0.17**	**0.11**
	S2	6.25 (0.97)	0.81	**0.13**	**−0.12**	**0.13**	0.08

Values highlighted in bold are significant at p < 0.001 level (two-tailed test).

The subscales of the MICC showed expected, mostly small magnitude but consistent with predictions associations, providing evidence for their construct validity. In addition, there were several medium to large magnitude associations, which we highlight in the following paragraphs. There were similarities and differences between undergraduate and open days students samples with differences reflecting tentative changes in motivation across years. However, the results must be interpreted with caution due to cross-sectional design of the study.

Helping demonstrated predicted positive correlation of large magnitude with the life goal Community in both samples, as well as positive correlation of medium magnitude with Personal Growth in the undergraduate sample. In addition, Helping demonstrated small magnitude positive correlations with Relationships, Agreeableness, Extraversion, Conscientiousness, and Openness in both samples. There was also a small magnitude correlation with Personal Growth in the potential students' sample. There were differences between samples: Helping demonstrated small magnitude negative correlation with the life goal Wealth in the potential students' sample, and a small positive with Fame in the undergraduate sample.

Loafing demonstrated only one medium magnitude correlation: a negative association with Agreeableness in the potential students' sample. The rest of the associations of Loafing with personality and life goals were of small magnitudes. Specifically, Loafing demonstrated predicted negative correlations with Extraversion and Conscientiousness; and the positive correlations with life goals of Wealth, Fame, and Image in both samples. In addition, Loafing correlated negatively with life goals Community, Personal Growth, and Relationships in both samples. It correlated negatively with Agreeableness in the undergraduate sample. There were differences between samples: Emotional Stability correlated negatively with Loafing only in the potential students' sample.

Conforming to the predictions, Interest demonstrated the predicted positive correlations of a medium magnitude with Personal Growth in both samples. In addition, Interest demonstrated predicted small magnitude correlations with Openness and Conscientiousness in both samples. There were small magnitude positive correlations with Agreeableness and life goals Community and Relationships in both samples. It also showed small size negative correlations with life goal Wealth in both samples. There were differences between samples. Interest showed the following additional small magnitude correlations: positive with Emotional Stability and Extraversion but only in the potential students' sample, and negative with life goal Image in the undergraduate sample.

Career demonstrated predicted positive medium magnitude correlations with the life goal Wealth in both samples, as well as small magnitude positive correlation with Conscientiousness. In addition, it correlated positively (small magnitude) with the life goals Fame, Image and Personal Growth, and Emotional stability and Openness, in both samples. There were differences between samples. Confirming predictions, Career correlated positively (small magnitude) with Extraversion, but only in the potential students' sample. In addition, Career correlated positively (small magnitude) with Agreeableness in the undergraduate sample. Career also positively correlated (small magnitude) with life goal Relationships in the undergraduate sample.

### Construct validity of the MICC scales

**Figure 5:**
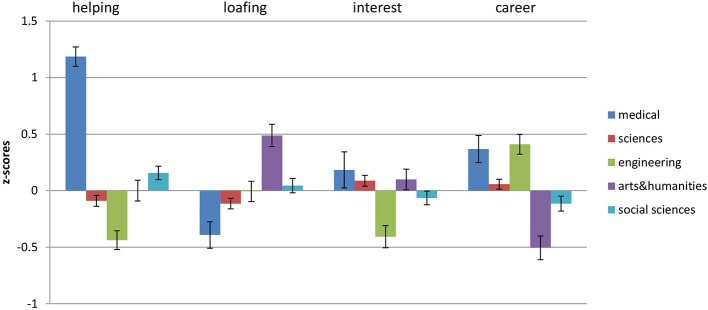
**Z-scores for Helping, Loafing, Interest and Career across all degree types in the undergraduate sample**. Error bars represent the standard error of the mean.

**Figure 6:**
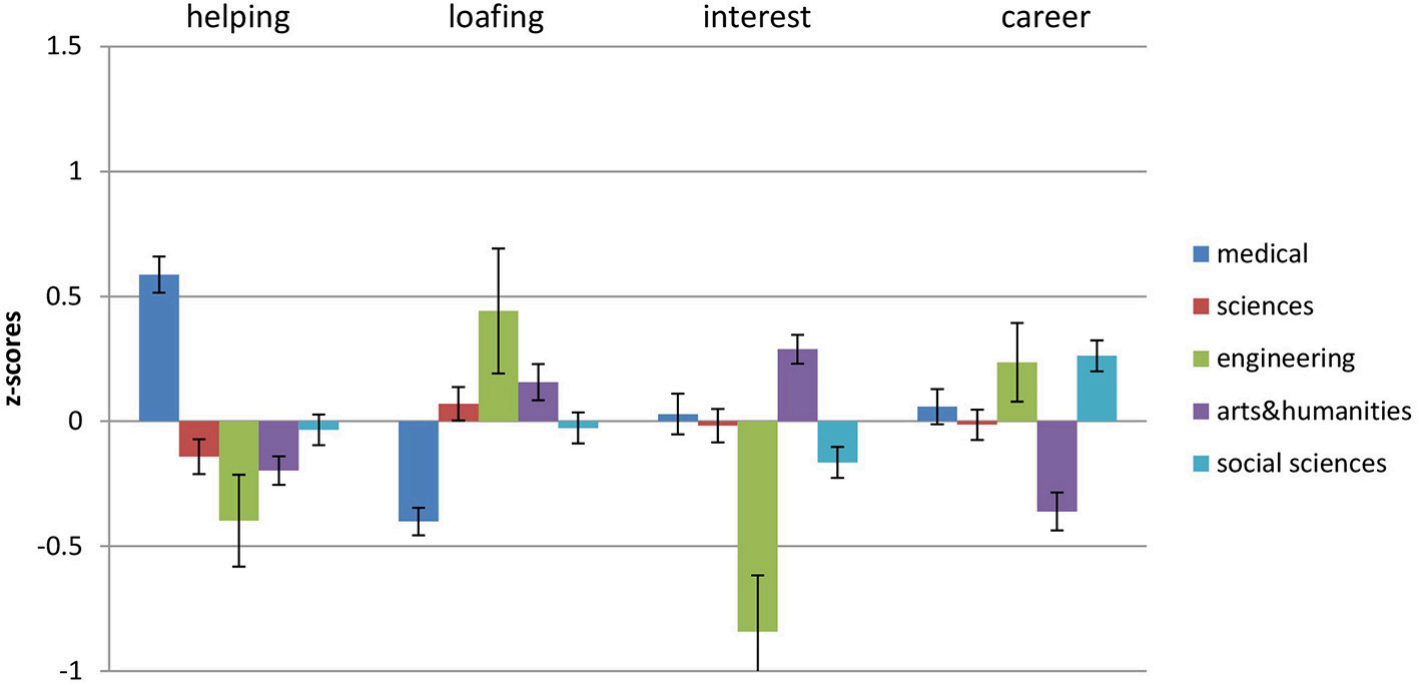
**Z-scores for Helping, Loafing, Interest and Career across all degree types in the potential students sample**. Error bars represent the standard error of the mean.

Using logistic regressions, we investigated how well specific dimensions of the MICC questionnaire predicted the choice of undergraduate degrees. A series of five hierarchical logistic regression tested predictive properties of the MICC scales with respect to undergraduate degree choice with age and gender controlled. The outcome variables for each regression were coded as 1 for the targeted degree type (e.g., medical sciences) vs. 0 for all other types. Age and gender were entered first, followed by life goals at Step 2, and the MICC scales at Step 3. We conducted additional analysis where each motivation was added separately at Step 3, and results are presented in Table 3. Steps 3a–d described in Table 3 estimated relative unique importance of each motivation for the choice of specific type of degree. The results are reported in Tables 4, 5 and Figures 5, 6.

**Table 4:** **Logistic regression models predicting degree types (Wald statistic, unstandardized *B*, odds ratio and respective confidence intervals for the MICC scales, Step 3 of regression analyses, see Table 5 for clarification), Sample 1 (S1, undergraduates, *N* = 844) and Sample 2 (S2, prospective students, *N* = 803)**.

**Degree type (outcome)**	**Variable**	**Sample**	**Wald statistic**	***B***	**Odds ratio**	**95% *CI* Lower**	**95% *CI* Upper**
Medical science Sample 1: *N* = 35 Sample 2: *N* = 145	Constant	S1	15.61^***^	−9.52			
		S2	0.48	−1.03			
	Helping	S1	24.51^***^	1.71	5.51	2.80	10.83
		S2	28.93^***^	0.70	2.01	1.56	2.58
	Loafing	S1	3.45	−0.55	0.64	0.37	1.11
		S2	11.60^**^	−0.67	0.51	0.35	0.75
	Career	S1	11.85^**^	0.87	2.39	1.45	3.92
		S2	3.68’	0.26	1.25	1.00	1.58
	Interest	S1	0.08	−0.08	0.83	0.51	1.35
		S2	3.48	−0.35	0.70	0.48	1.02
Sciences Sample 1: *N* = 375 Sample 2: *N* = 217	Constant	S1	0.11	0.30			
		S2	2.24	2.95			
	Helping	S1	9.90^**^	−0.26	0.77	0.65	0.91
		S2	3.92^*^	−0.18	0.83	0.70	1.00
	Loafing	S1	2.11	−0.14	0.87	0.72	1.05
		S2	0.88	0.12	1.13	0.88	1.45
	Career	S1	8.18^**^	0.22	1.24	1.07	1.44
		S2	0.11	1.31	1.11	0.93	1.33
	Interest	S1	5.11^*^	0.18	1.20	1.02	1.41
		S2	0.29	0.08	1.08	0.81	1.44
Engineering Sample 1: *N* = 97 Sample 2: *N* = 25	Constant	S1	0.03	0.16			
		S2	0.28	1.54			
	Helping	S1	12.23^**^	−0.50	0.61	0.46	0.80
		S2	0.09	−0.08	0.93	0.56	1.54
	Loafing	S1	1.86	−0.13	0.84	0.61	1.16
		S2	0.34	0.18	1.20	0.65	2.21
	Career	S1	25.01^***^	0.74	2.09	1.57	2.79
		S2	4.68^*^	0.60	1.83	1.06	3.17
	Interest	S1	−0.44^**^	−0.44	0.65	0.50	0.83
		S2	9.10^**^	−1.01	0.36	0.19	0.70
Arts and humanities Sample 1: *N* = 106 Sample 2: *N* = 189	Constant	S1	2.38	−2.95			
		S2	24.55^***^	−8.10			
	Helping	S1	0.08	0.04	1.04	0.79	1.36
		S2	11.03^**^	−0.34	0.71	0.58	0.87
	Loafing	S1	26.28^***^	0.76	2.15	1.60	2.88
		S2	12.06^**^	0.51	1.67	1.25	2.22
	Career	S1	30.08^***^	−0.66	0.51	0.41	0.65
		S2	36.30^***^	−0.63	0.53	0.44	0.66
	Interest	S1	9.08^**^	0.46	1.58	1.17	2.12
		S2	26.81^***^	1.05	2.85	1.92	4.24
Social science Sample 1: *N* = 226 Sample 2: *N* = 212	Constant	S1	0.85	−0.97			
		S2	0.06	0.30			
	Helping	S1	15.05^***^	0.37	1.45	1.20	1.75
		S2	0.06	0.02	1.02	0.85	1.23
	Loafing	S1	0.07	−0.03	0.97	0.79	1.19
		S2	7.44^**^	−0.37	0.69	0.52	0.90
	Career	S1	18.81^***^	−0.36	0.70	0.59	0.82
		S2	7.03^**^	0.26	1.29	1.07	1.57
	Interest	S1	3.79’	−0.17	0.84	0.71	1.00
		S2	8.76^**^	0.30	0.66	0.50	0.87

Note.

*** p < 0.001,

** p < 0.01,

* p <.05,

’ p = 0.05

**Table 5:** **Fit statistics of logistic regression models predicting degree types, Sample 1 (undergraduates) and Sample 2 (prospective students): Step 1, age and gender; Step 2, Aspiration Index scales; Step 3, MICC scales**.

**Degree type (outcome)**		**Undergraduates**		**Prospective students**	
		**Step's χ ^**2**^**	**Nagelkerke *R*^2^**	**Step's χ ^**2**^**	**Nagelkerke *R*^2^**
Medical degrees vs. other degrees	Step 1	5.23	0.021	2.10	0.004
	Step 2	33.31^***^	0.132	58.15^***^	0.114
All four motivations	Step 3	57.27^***^	0.215	57.17^***^	0.105
	Overall model	95.82^***^	0.368	117.42^***^	0.223
Helping	Step 3a	40.71^***^	0.154	38.22^***^	0.071
Loafing	Step 3b	1.79	0.007	10.79^**^	0.021
Career	Step 3c	19.52^***^	0.072	11.43^**^	0.022
Interest	Step 3d	1.25	0.005	0.77	0.002
Sciences vs. other degrees	Step 1	18.67^***^	0.029	8.08^*^	0.015
	Step 2	7.20	0.012	13.47^*^	0.023
All four motivations	Step 3	21.35^***^	0.033	5.28	0.01
	Overall model	47.82^***^	0.074	26.84^***^	0.048
Helping	Step 3a	5.38^*^	0.008	2.75	0.028
Loafing	Step 3b	3.19	0.004	0.78	0.002
Career	Step 3c	5.39^*^	0.008	0.56	0.001
Interest	Step 3d	4.11^*^	0.006	0.08	0.001
Engineering vs. other degrees	Step 1	81.65^***^	0.181	26.70^***^	0.150
	Step 2	17.12	0.036	11.74	0.058
All four motivations	Step 3	51.12^***^	0.102	13.58^**^	0.065
	Overall model	149.9^***^	0.319	55.03^***^	0.273
Helping	Step 3a	10.45^**^	0.021	0.03	0
Loafing	Step 3b	2.02	0.004	1.25	0.006
Career	Step 3c	20.11^***^	0.041	4.24^*^	0.02
Interest	Step 3d	13.08^***^	0.027	7.65^**^	0.037
Arts and humanities vs. other degrees	Step 1	9.85^**^	0.023	3.86	0.007
	Step 2	20.22^**^	0.046	35.05^***^	0.064
All four motivations	Step 3	62.22^***^	0.136	94.88^***^	0.160
	Overall Model	92.29^***^	0.205	133.79^***^	0.231
Helping	Step 3a	0.15	0.001	15.72^***^	0.028
Loafing	Step 3b	22.32^***^	0.05	6.23^*^	0.011
Career	Step 3c	26.81^***^	0.06	45.76^***^	0.08
Interest	Step 3d	5.94^*^	0.013	24.93^***^	0.044
Social sciences vs. other degrees	Step 1	1.09	0.002	2.13	0.004
	Step 2	11.04	0.019	23.36^**^	0.042
All four motivations	Step 3	30.59^***^	0.050	21.21^***^	0.037
	Overall model	42.48^***^	0.071	46.71^***^	0.083
Helping	Step 3a	6.81^**^	0.011	0.21	0
Loafing	Step 3b	0.017	0	5.54^*^	0.009
Career	Step 3c	13.05^***^	0.021	6.19^*^	0.01
Interest	Step 3d	2.36	0.003	6.52^*^	0.011

Steps 3a–d represent additional analysis, where instead of adding all four motivations, only one motivation at a time was added to a regression model.

Note.

*** p < 0.001,

** p < 0.01,

* p < 0.05,

’*p = 0.05.*

In line with predictions, the choice of medical degrees was positively associated with Helping and Career motivation in both samples. In both samples Helping motivation explained the highest proportion of variance compared to other motivations. In addition, medicine was negatively associated with Loafing in the potential students' sample. In both samples the choice of science degree was negatively associated with Helping. In addition, in the undergraduate sample, the choice of science degree was positively associated with Interest and Career. There was little difference in the relevant contribution each motivation has made to the explanation of the choice of science degree. The choice of engineering degree was positively associated with Career and negatively with Interest in both samples. In addition, engineering undergraduates reported Helping as low in importance in terms of their degree choice. For the undergraduate sample only, Career was the strongest predictor over other motivations in terms of explained variance. There was no consistency between samples in terms of predictions for social science degrees. Those who were studying social sciences reported Helping as important, and Career and Interest as unimportant reasons for their degree choice, with Career motivation explaining the most of the variance. Those who were planning to study social sciences reported Career and Interest as important when considering the choice of degree, at the same time being low on Loafing. Current and potential students, who chose/were choosing to study arts and humanities degrees, were concerned how interesting the degree was for them (high Interest) and how easy it would be to complete it (high Loafing), while not taking into account career prospects (low Career). In addition, potential students who were planning to study arts and humanities, reported low concern about opportunities to help others (low Helping). In terms of variance explained, for undergraduate students Loafing (high) and Career (low) were the strongest predictors, while for potential students the strongest predictors were Career (low) and Interest (high).

### Incremental validity of the MICC scales

Finally, we tested incremental validity of the MICC scales over the life goals with respect to undergraduate degree choice using the results of the regressions described above (Table 5). Age and gender significantly predicted all degree types apart from social and medical sciences. Life goals added significantly to explaining variance for medical sciences and arts and humanities. The addition of the MICC also added significantly to explaining the variance in degree choice for all degree types. Thus, degree choice motivations demonstrated incremental validity over general life goals.

## Discussion

The 18-items MICC questionnaire is a new measure of individual differences in undergraduate degree choice motivation. We identified four subscales of the MICC that equate to four domains of motivation: Career, Interest, Loafing, and Helping. The results indicate that the MICC has a clear factor structure and strong psychometric properties. Different subscales of the MICC questionnaire showed predicted associations with life goals and the Big Five domains.

Both Helping and Interest correlated positively with all three intrinsic life goals (Personal Growth, Community, and Relationships), suggesting that these subscales capture concern for others as well as striving for personal growth, as defined by SDT (Kasser and Ryan, 1996). In terms of associations between personality traits and the MICC scales, a few interesting patterns have emerged. Helping and Interest were again associated with the same personality traits: Agreeableness, Conscientiousness, and Openness. In addition, Helping and Interest had highest inter-scales correlations. However, Helping was psychometrically distinct from Interest, and predicted different choices of degree: Helping consistently, across two samples, predicted the choice of medical degree, while Interest predicted the choice of arts and humanities. On the one hand this supports SDT, suggesting that pro-social and intrinsic motivations are linked, as they were related to the same personality traits. However, this also suggests that when facing a specific choice, those who want to help others make different decisions compared to those who are focused on intrinsic enjoyment. It is possible that in cases of some degrees (e.g., arts), decision will be driven by higher intrinsic motivation or expectations to enjoy the degree but pro-social motivation would not be a consideration. Thus, these motivations, while associated with the same personality traits, result in different outcomes.

Further, both Loafing and Career motivations were related positively with a set of extrinsic life goals (Wealth, Fame, and Image) confirming that Loafing and Career are aligned with extrinsic motivation. However, they were distinct psychometrically and were associated with different degrees choices. Career, consistently across both samples, positively predicted the choice of medical and engineering degrees, while Loafing predicted the choice of arts and humanities. In addition, Career was positively associated with intrinsic life goals: Personal Growth in both samples and Relationships in the undergraduate sample. Loafing was negatively associated with all three intrinsic life goals: Personal Growth, Relationships, and Community. Furthermore, in terms of personality traits, Loafing was associated negatively with Extraversion, Agreeableness and Conscientiousness, while Career demonstrated an opposite pattern. Thus, those who indicated Loafing as a motivation for their degree choice were less sociable, and were less friendly and more disorganized. Those who indicated Career as a motivation for their degree choice were sociable, friendly, and organized, plus emotionally stable and opened to new experiences.

These findings add to the previous theoretical literature on motivation. SDT proposes that intrinsic and extrinsic motivations are the different end of the same continuum, and measuring individual differences on this continuum is sufficient to predict real life outcomes. In our study both intrinsic and extrinsic dimensions were represented by two motivations each: Career and Loafing for extrinsic, and Helping and Interest for intrinsic. Further, all four undergraduate degree choice scales were psychometrically distinct. Based on SDT, we should be able to predict real life outcomes from the extent to which a person is extrinsically or intrinsically motivated. However, it seems that one dimension might be not enough to make a prediction about specific outcome and, in addition, both intrinsic and extrinsic motivations have different expressions. For example, somebody who is intrinsically motivated could have been driven by interest in the subject and chose to study arts, while if they were striving to help others, they might have chosen to study medicine. Furthermore, as our data shows, it is plausible that both an intrinsic and extrinsic type of motivation could drive the choice of the same individual. For example, medical degrees were chosen because of Helping and Career. This is in line with Chemolli and Gagné (2014) who used Rasch analysis to verify the unidimensional continuum of intrinsic motivation using two SDT scales (the Multidimensional Work Motivation Scale and the Academic Motivation Scale) and found that multidimensional conceptualization of motivation might represent the structure of human motivation better. Our study contributes to these results, further suggesting four different motivational dimensions that can better explain real life choices than unidimensional models of motivation.

Our data supports the multideterministic nature of the choice (Pervin, 2001). We demonstrated that medical students chose their degrees because of career prospects and opportunity to help others, while engineering students strive for good career opportunities and regard intrinsic interest in the subject as an unimportant factor in the choice of degree. Further, science students reported low concern about opportunities to help others in their future career (across both samples) and high Interest (undergraduates), while arts and humanities students reported high Interest combined with low career concerns and high Loafing. Finally, the motivations of social science students were inconsistent between samples, which might reflect differences in subjects that were included in each sample in social science category. Future studies could investigate undergraduate degree choice reasons for choosing specific subjects, such as law vs. social work.

Theoretically this study contributes a set of “proximal” motivational constructs, which can explain differences in choices within a specific context of career path. Proximal motivations have been discussed in previous research and are more contextualized in relation to choice, compared to generic motivations. Previous researchers discussed the need to measure proximal motivation in addition to more distal constructs, such as life goals and personality traits, in order to improve predictability of the models (Elliot, 2006). The current study theoretically identified and empirically tested four motivations for choosing a degree: Interest (motivated by the enjoyment of the activity), Career (motivated by achievement striving), Loafing (led by the choice of easy options), Helping (motivated by benefit to others). It is plausible that there is consistency in motivation (e.g., to help) across domains (e.g., medicine as a degree, volunteering as an extra curriculum activity). Indeed, there is some evidence that social preferences for cooperation are stable (Volk et al., 2012). Future studies could investigate if there is consistency in people's proximal motivations in different domains or the motivations are context and domain specific.

In addition to differences in personality and life goals, we reported gender differences in degree choice motivation. Male participants reported higher Career (only Sample 1) and Loafing motivation (both samples), while female participants were high in Helping (both samples) and Interest (only Sample 1, with the effects of gender on motivation Sample 2 explained by personality traits, see the footnote). These findings support previous research in gender specific social roles in terms of Helping and Career, however, it is not clear why there should be gender difference in Interest and Loafing. Further research could investigate whether gender stereotypes predict the choice of undergraduate degree or whether the differences are mediated by other factors, such as the type of chosen degree.

The information about what motivates students to choose their degree can be potentially very important for educational institutions and individual departments. For example, it is important to recognize that engineering students currently may not be as concerned about the enjoyment they can gain from studying their future undergraduate degree as lack of interest in the subject leads to the low levels of deep learning (McManus et al., 1998). This can in turn impact understanding of the material and student satisfaction. Therefore, establishing low levels of intrinsic motivation in engineering students might prompt departments to pay more attention to getting students to engage in the process of learning and enhance their intrinsic motivation and interest in the subject. Further, the fact that arts and humanities students reported significantly higher levels of Loafing than other students can be used as a trigger for respective departments to focus more on societal impact of the future careers in arts and humanities. This can contribute to the quality of students' education making their future work endeavors more relevant to societal needs.

Our study had some limitations. The current study used a cross-sectional design, which does not allow determining whether the motivation for undergraduate degree choice predicts their actual choice. In our prospective students' sample, participants stated the degree they wished to choose to study at the university, which might not correspond to the one they ended up taking, while degree reasons reported by undergraduates could have been biased by the experience during their current education. Longitudinal research is needed to understand developmental trajectories of different motivations, and their associations with real life choices. Further, there might be some types of degrees, which combine two different degree type areas, such as science and humanities (e.g., BA in Psychology and Philosophy). Future studies could benefit by looking into specific subsample of degree types to investigate whether MICC questionnaire holds predictive power for these specific degrees. Finally, we are aware that not all countries' educational systems require students to choose their undergraduate degree prior to the start of their time at university; however it is a common situation in many countries (e.g., UK, Germany, Russia).

Overall, the current results demonstrate that individual differences in motivation (Helping, Career, Interest, and Loafing) are associated with real life choices in the context of the choice of undergraduate degree. The implications of distinct types of contextualized proximal motivation for predicting life changing choices are an exciting research avenue. After establishing the dimensions, it is possible to investigate if those proximal motivations mediate the effects of more distal dispositional motivations (such as approach and avoidance) and personality dispositions on the choice of degree. It will help to establish link between motivational and personality constructs of different levels and actual choices. This will result in better understanding and improved predictions of differences in real life choices.

### Conflict of interest statement

The authors declare that the research was conducted in the absence of any commercial or financial relationships that could be construed as a potential conflict of interest.

## Footnotes

^1^The gender differences in motivations could have been due to differences in personality. For instance, it was demonstrated that females are generally higher in Agreeableness and Conscientiousness (e.g., Schmitt et al., 2008), which could in turn explain differences in Helping and Loafing motivations. We conducted additional analysis to check this hypothesis. First, females were higher in Agreeableness [S1: *t*(813) = −2.97, *p* < 0.005, *d*= 0.21 (0.07; 0.35); S2: *t*(813) = −4.12, *p*< 0.005, *d*= 0.32 (0.77; 0.47)] and Conscientiousness [S1: *t*(813) = −4.87, *p*< 0.001, *d*= 0.35 (0.21; 0.49); S2: *t*(813) = −3.41, *p*< 0.005, *d*= 0.27 (0.11; 0.42)], and lower in Emotional Stability [S1: *t*(813)= 5.56, *p*< 0.001, *d*= 0.40 (0.25; 0.54); S2: *t*(813) = −4.16, *p*< 0.001, *d*= 0.32 (0.17; 0.48)] than men, with no gender differences for Extraversion and Openness. Second, a series one-way between subjects ANOVAs showed that the effects of gender on motivation remained significant when controlling for personality traits, except for Career and Interest in the open days sample (consistent with analysis reported in the main body of the paper): Helping [S1: *F*(1, 808) = 11.6, *p*< 0.005; S2: *F*(1, 808) = 8.67, *p*< 0.005], Career [S1: *F*(1, 808) = 17.99, p< 0.001; S2: F(1, 808) = 0.72, *p* = 0.395], Interest [S1: *F*(1, 808) = 14.88, *p*< 0.001; S2: *F*(1, 808) = 2.15, *p*= 0.14] and Loafing [S1: *F* (1, 808) = 18.58, *p*< 0.001; S2: *F*(1, 808) = 6.45, *p*< 0.05].
