# Supplementary material for: Why do different people choose different university degrees? Motivation and the choice of degree
Source: Front Psychol. 2014 Nov 13;5:1244. doi: 10.3389/fpsyg.2014.01244 (PMC4230040; doi:10.3389/fpsyg.2014.01244)
Supplement: Supplementary file 1 [file Table1.DOCX]

Supplementary materials.

Table S1. Initial pool of items for the MICC questionnaire. The items which remain in the final version are labelled with respective numbers.

| Item Number in a  final version of the questionnaire | I have chosen this degree because…. |
| --- | --- |
|  | ….I wouldn’t want to do a job that involves hurting other people’s feelings. |
|  | ….I want to realize my goals at any costs, even when at the expense of others. |
| 03 | ….I’m not particularly concerned about other people. |
| 05 | ….my individual goals are more important than the prosperity of society. |
| 06 | ….it was the easiest option for me. |
|  | ….I want to be in a position to get people to do want I want them to do. |
|  | ….I enjoy manipulating other people’s feelings. |
| 14 | ….I knew that I’d manage to pass the degree without doing too much work. |
|  | ….I want to influence other people’s actions. |
| 16 | ….the degree seemed to be easy to pass. |
|  | ….I am good at telling people what they want to hear, in order to get them to do what I want. |
| 01 | ….I was always interested in this subject. |
| 04 | ….I wanted to know more about this subject. |
| 11 | ….for me it is very important to study a degree that I enjoy. |
|  | ….it’s an enjoyable subject to study. |
| 13 | ….it is a fascinating subject to study. |
|  | ….when I found out about this course, I instantly knew that I want to apply: It was one-moment decision. |
|  | ….it was something new and exciting. |
|  | ….I feel it will lead to personal development. |
|  | ….it was interesting, even though it doesn’t provide very secure career options. |
|  | ….I don’t want to deal with other people’s feelings. |
|  | ….I would prefer to have a job where I don’t need to interact with people a lot. |
| 02 | ….I want to help other people. |
|  | ….in my future job I want to interact with people as much as possible. |
| 09 | ….I want to serve society. |
| 12 | ….I am interested in people. |
|  | ….I will feel more comfortable when working with people. |
|  | ….the call of duty means a lot to me. |
| 17 | ….I am interested in understanding other people’s perspectives. |
| 18 | ….I want to make world a better place. |
|  | ….I didn’t know what else to do. |
|  | ….I didn’t care that much about what I’m going to study. |
|  | ….I avoid highly competitive situations, and getting into this program was not very competitive. |
|  | ….a particular lifestyle is important for me. |
|  | ….I was good at this subject at school. |
|  | ….I want to be respected by other people. |
|  | ….it’s intellectually challenging. |
| 07 | ….I want to get a well-paid job in the future. |
| 08 | ….it is very competitive and I am an achiever. |
| 10 | ….because it provides good career options. |
|  | ….I knew exactly what I would get from the course. |
|  | ….I carefully appraised all pros and cons and this degree was the best one for me. |
| 15 | ….it provides me with secure career options. |
|  | ….a high social status is important for me. |
|  | ….it was a safe option for me. |
|  | ….because according to my extensive research of various opportunities, it was the best option. |
|  | ….of family influences. |
|  | ….I didn’t want to move far from family and/or friends. |
|  | ….of the influence of my friend(s). |
|  | ..... of a school teacher suggestion |
|  | ....I was always interested in this subject |
|  | .... I wanted to live in Nottingham: I like the city |
|  | ….of pure chance. |
|  | ….I didn’t get in where I wanted to go. |
|  | ….it was the only offer I had. |
